# Nuclear Magnetic Resonance of Hydrogen Molecules Trapped inside C_70_ Fullerene Cages

**DOI:** 10.1002/cphc.201300269

**Published:** 2013-06-20

**Authors:** Salvatore Mamone, Maria Concistrè, Ivo Heinmaa, Marina Carravetta, Ilya Kuprov, Gary Wall, Mark Denning, Xuegong Lei, Judy Y-C Chen, Yongjun Li, Yasujiro Murata, Nicholas J Turro, Malcolm H Levitt

**Affiliations:** [a] School of Chemistry, Southampton University Southampton SO17 1BJ (United Kingdom); [b] National Institute of Chemical Physics and Biophysics Akadeemia tee 23, 12618 Tallinn (Estonia); [c] Department of Chemistry, Columbia University New York, New York 10027 (USA); [d] Institute for Chemical Research, Kyoto University Uji, Kyoto 611-0011 (Japan)

**Keywords:** C_70_, cryogenic nmr spectroscopy, endofullerenes, quantum dynamics, solid-state nmr spectroscopy

## Abstract

We present a solid-state NMR study of H_2_ molecules confined inside the cavity of C_70_ fullerene cages over a wide range of temperatures (300 K to 4 K). The proton NMR spectra are consistent with a model in which the dipole–dipole coupling between the *ortho*-H_2_ protons is averaged over the rotational/translational states of the confined quantum rotor, with an additional chemical shift anisotropy *δ*^H^_CSA_=10.1 ppm induced by the carbon cage. The magnitude of the chemical shift anisotropy is consistent with DFT estimates of the chemical shielding tensor field within the cage. The experimental NMR data indicate that the ground state of endohedral *ortho*-H_2_ in C_70_ is doubly degenerate and polarized transverse to the principal axis of the cage. The NMR spectra indicate significant magnetic alignment of the C_70_ long axes along the magnetic field, at temperatures below ∼10 K.

## 1. Introduction

**Figure 1:**
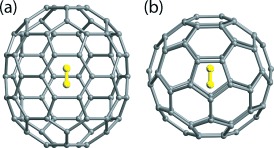
The endohedral fullerene H_2_@C_70_ shown in a) equatorial and b) polar views. The carbon cage and the endohedral H_2_ are shown by a ball-and-stick representation.

Fullerenes consist of symmetrical carbon-only cages surrounding a nanoscale cavity.^1^ Synthetic routes have been developed for inserting small molecules such as H_2_ and H_2_O into the cavity, which may then be resealed.^2^–^4^ This “molecular surgery” procedure has been successfully completed on C_70_, which consists of 70 carbon atoms arranged in an ellipsoidal shape resembling a rugby ball, with point group symmetry *D*_5*h*_. In the resultant product, most of the fullerenes encapsulate one hydrogen molecule, obeying the formula H_2_@C_70_ (Figure 1), although there is a small percentage, about 3 %, of doubly occupied cages.^5^

The endohedral hydrogen molecules behave as molecular quantum rotors and exhibit quantization of all motional degrees of freedom (vibrations, rotations and translations).^6^, ^7^ The homogeneity of the trapping sites and the relative isolation of the guest molecules make these systems excellent targets for solid-state spectroscopic studies^8^–^15^ and quantum mechanical calculations.^16^–^18^ Comparison with high-resolution spectroscopic data allows refinement of the parameters for the non-bonded interaction of the hydrogen molecule with the carbon surface.^13^, ^19^

H_2_ also displays spin isomerism. According to the Pauli principle the molecular wave function is antisymmetric with parity −1 with respect to the exchange of the two identical protons. In the electronic ground state of H_2_ the parity of the molecular wave function is (−1)^(*I*+*J*)^ where *I* is the nuclear spin and *J* is the angular momentum quantum number for rotations around the center of mass. The nuclear spin singlet (*I*=0, *para*-H_2_) is combined with even-*J* functions, while the nuclear spin triplet (*I*=1, *ortho*-H_2_) is combined with odd-*J* functions. The small moment of inertia leads to a large separation between the rotational energy levels of free H_2_, while the spin mixing terms are relatively small. As a result, the interconversion between spin isomers is slow in the absence of an external spin catalyst. Spin-isomer conversion in dihydrogen endofullerenes has been induced by molecular oxygen,^20^, ^21^ covalently linked magnetic switches^22^ and by photo-excitation of electronic triplet states.^23^

In this paper we report ^1^H NMR lineshapes and spin-lattice relaxation times *T*_1_ for a powder sample of H_2_@C_70_. The ^1^H NMR spectra display temperature-dependent lineshapes, with no evidence of *ortho*–*para* conversion for the endohedral H_2_ molecules. The lineshapes are consistent with a model in which the dipole–dipole coupling between the nuclei is averaged over the accessible translational–rotational wavefunctions and in which there is also a significant chemical shift anisotropy (CSA) interaction. The proton CSA is found to be almost temperature-independent and is attributed to the effect of the carbon cage. This conclusion is supported by DFT calculations of the chemical shift tensor field inside the cage.

The confinement potential of H_2_ inside C_70_ reflects the *D*_5*h*_ point-group symmetry of the cage. The non-spherical symmetry leads to a splitting of the three-fold degenerate *ortho*-H_2_ rotational ground state into two levels with degeneracies 1 and 2. Studies of the five-dimensional quantum mechanics of H_2_ in a modelled cage potential have predicted that the ground state is non-degenerate, while the upper sublevel is doubly degenerate.^19^ However, as discussed below, the experimental NMR data indicate that the ground state of *ortho*-H_2_ in C_70_ is two-fold degenerate, with a non-degenerate upper sublevel. This energy ordering is consistent with recent infrared spectroscopic data.^24^

An unexpected effect is detected in the ^1^H NMR spectra of H_2_@C_70_ at temperatures below ∼10 K. Changes in the lineshape indicate significant alignment of the C_70_ long axes along the magnetic field, indicating that either the C_70_ molecules rotate on their crystal lattice points to align along the magnetic field, or possibly that entire domains or crystallites reorient with the field. These results show that the endohedral hydrogen molecules may act as low-temperature “NMR indicators” which report on the behaviour of the enclosing carbon cages.

## Materials and Methods

### Sample

H_2_@C_70_ was synthesized via “molecular surgery” according to the method of Komatsu and Murata.^3^, ^5^ A sufficiently large orifice was opened in each C_70_ cage by a series of controlled reactions. Molecular hydrogen was forced into the open cages and remained trapped when ordinary conditions were restored. The holes were sealed by another series of chemical reactions without escape of the hydrogen. High-performance liquid chromatography was used to remove the residual empty fullerenes leaving a sample with ∼100 % of the fullerenes filled.

About 25 mg of H_2_@C_70_ was dissolved in 3 mL of CS_2_. H_2_@C_70_ was precipitated by adding the solution to 50 mL of pentane while stirring, and then centrifuging. The precipitated H_2_@C_70_ was separated and heated at 60 °C in vacuum for 3 days, at 80 °C for 3 more days and at 180 °C for further 3 days. All results were obtained on 3 mg of H_2_@C_70_ in a low-proton-content Pyrex tube, evacuated for approximately 1 hour at 80 °C and flame-sealed.

### NMR Experiments

**Figure 2:**
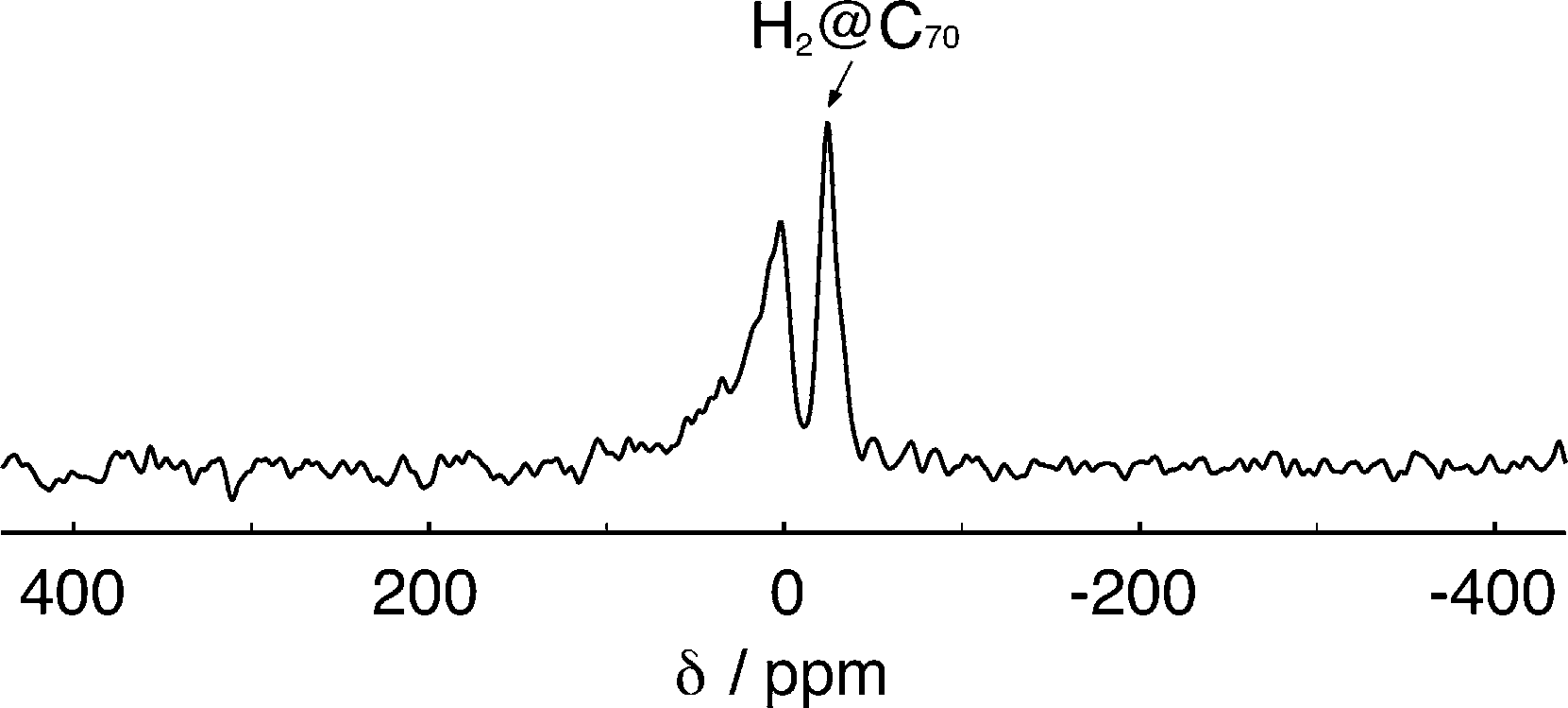
^1^H NMR spectrum of a solid sample of H_2_@C_70_ at room temperature in a magnetic field of 14.1 T, acquired under static conditions (i.e. without sample rotation). The spectrum was obtained by the Fourier transformation of NMR signals induced by 90° radio-frequency pulse. The chemical shift scale was calibrated by setting the peak of adamantane in the solid phase at 1.8 ppm. The chemical shift of the H_2_@C_70_ peak is −24.7 ppm while the signal of the protonated impurity has a maximum at 1.2 ppm.

The proton spectrum of the static sample, shown in Figure 2, was obtained at room temperature in a magnetic field of 14.1 T using a Bruker AVANCE-II+ spectrometer in Southampton and a home-built NMR probe with a solenoid coil. A single 90° pulse with duration 4.5 μs was used to excite the proton free induction decay. The NMR signal was detected after a ring-down delay of 5 μs. The spectrum is an average of 4 transients with an inter-pulse delay of 5 s.

All other NMR data were obtained using a high-field FT-NMR spectrometer with a magnetic field of 8.5 T at the National Institute of Chemical Physics and Biophysics in Tallinn (Estonia). The instrumental apparatus is partly described in refs. 8, 9. The experiments were performed on a static sample using a home-built cryogenic NMR probe with a radiofrequency solenoid coil perpendicular to the static magnetic field. The rf fields gave rise to a ^1^H nutation frequency of ∼250 kHz. The temperature was monitored by using a calibrated LakeShore Cernox sensor placed close to the sample and controlled with an accuracy of ±0.1 K down to 4.3 K.

**Figure 3:**
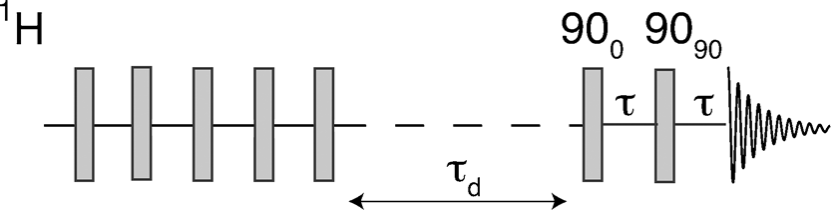
Pulse sequence used for variable-temperature ^1^H NMR on H_2_@C_70_. A comb of 90° pulses is used to saturate the magnetization, followed by a variable delay *τ*_d_ for recovery of the longitudinal magnetization. A solid echo sequence composed of two 90° pulses, with a relative phase shift of 90°, refocusses the inhomogeneous dephasing caused by the intramolecular dipole–dipole interaction for isolated spin-1/2 pairs. Signal acquisition is started at the top of the echo.

The pulse sequence for acquisition of the variable-temperature NMR data is shown in Figure 3. This consisted of a saturation comb, a variable recovery delay *τ*_d_, and a solid echo sequence of two 90° pulses, followed by acquisition of the free-induction decay (FID). The saturation comb ensured a reproducible initial condition for each NMR pulse sequence and consisted of 120 90° pulses separated by delays of 500 μs. All 90° pulses had a duration of between 1.2 and 0.9 μs, which ensured approximately uniform excitation over the spectral bandwidth.

The solid echo block consisted of two 90° pulses with a relative phase shift of 90°, separated by a delay of 100 μs. Signal acquisition was initiated 100 μs after the second pulse. In the case of isolated homonuclear 2-spin-1/2 systems, in which the linewidth is dominated by the orientation-dependent intramolecular dipole–dipole interaction, the strong inhomogeneous decay of the NMR signal between the two 90° pulses is accurately reversed after the second pulse. Observation of the signal at the peak of the spin echo therefore allowed observation of the rapidly decaying initial part of the free-induction decay, while partially suppressing the signals from protonated impurities, which do not refocus accurately, since they do not originate with isolated spin pairs. This procedure was used before in the study of endohedral hydrogen-fullerene complexes.^8^, ^9^

The integrated area of the NMR signals was monitored as a function of the recovery delay *τ*_d_, and fitted to an exponential function, in order to determine the spin-lattice relaxation time constant *T*_1_. Typically, 32 experiments were recorded for each *T*_1_ recovery curve. After making rough estimates of *T*_1_ through preliminary studies, fully relaxed NMR spectra were obtained by using recovery delays *τ*_d_ which are long compared to *T*_1_.

### Computational Methods

**Figure 4:**
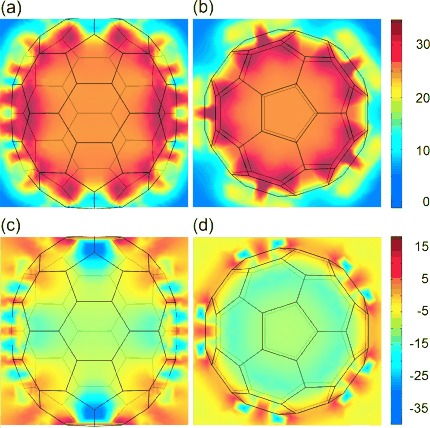
Longitudinal and equatorial plane cross-sections of absolute chemical shielding tensor field (in ppm, computed using GIAO DFT M06/cc-pVDZ method in Gaussian09) inside an empty C_70_ cage at the Born–Oppenheimer energy minimum geometry. Upper panels: isotropic chemical shielding *σ*_iso_=(*σ_XX_*+*σ_YY_*+*σ_ZZ_*)/3; lower panels: chemical shielding anisotropy *σ*_CSA_=*σ_ZZ_*−*σ*_iso_, where *Z* indicates the long axis of the cage.

The absolute chemical shielding tensor field inside the C_70_ cage (Figure 4) was calculated using the following procedure in Gaussian 09.^25^ The energy minimum geometry was obtained with the DFT M06/cc-pVDZ method. GIAO DFT M06/cc-pVDZ calculations of chemical shielding tensors were then performed repeatedly for 2500 randomly selected positions of a ghost atom inside and outside the cage, corresponding to 50 000 spatial data points after taking into account the *D*_5*h*_ symmetry of the cage. The shielding tensors were then interpolated using cubic splines onto a flat 3D grid (200 points in each dimension) and the resulting tensor field cubes were used for the average shielding tensor estimates and plotting.

The magnetic susceptibility tensor of a C_70_ molecule was calculated for an empty cage at four different levels of theory (GIAO M06/cc-pVDZ, GIAO M06/cc-pVTZ, CSGT M06/cc-pVDZ, CSGT M06/cc-pVTZ) in Gaussian 09, with similar results for the eigenvalues of the susceptibility tensor (to within 10 %) in all cases. The choice of the M06 exchange-correlation functional^26^ in all calculations is dictated by its superior performance with dispersion interactions that are expected to be significant in an extended and strained aromatic system such as the C_70_ fullerene cage. Another reason is relatively accurate excited state energies, which determine the accuracy of magnetic shielding calculations because they appear in perturbation theory denominators.^27^

After the shielding tensor field was obtained, the average anisotropy of the chemical shielding induced by the cage was obtained as follows: the spatial volume accessible to the hydrogen molecule inside the fullerene cage was estimated by taking the difference between the geometrical volume of the cage and the union of the carbon atom spheres with an assigned van der Waals radius of 1.70 Å. The average of the chemical shielding tensor field, obtained from DFT calculations as described above, was calculated over the resulting “accessible” volume and its anisotropy computed as a difference between the component along the axis of the cage and the isotropic component.

## 2. Results

### 2.1. Room-Temperature NMR

The room-temperature ^1^H spectrum of the static sample, obtained by a single 90° pulse, is shown in Figure 2. This displays two resolved peaks at −24.7 ppm and +1.2 ppm, with an amplitude ratio of 1:2. The narrow −24.7 ppm peak is assigned to the endohedral protons of H_2_@C_70_, since the unusual chemical shift is similar to the −23.97 ppm shift for H_2_@C_70_ in solution.^5^ The broader signal at +1.2 ppm is attributed to protonated impurities, probably from occluded solvent molecules. It was verified that this peak is not due to background signals from the probe or the Pyrex tube. We could not resolve any signals from doubly-occupied C_70_ cages, which are observed in solution at −23.80 ppm.^5^

It is unusual to obtain resolved proton spectra in the solid state, without the assistance of magic-angle spinning or homonuclear dipolar decoupling. The strong negative chemical shift of the endohedral H_2_ molecules, and the low proton density, make this sample a special case.

### 2.2. Variable-Temperature NMR

**Figure 5:**
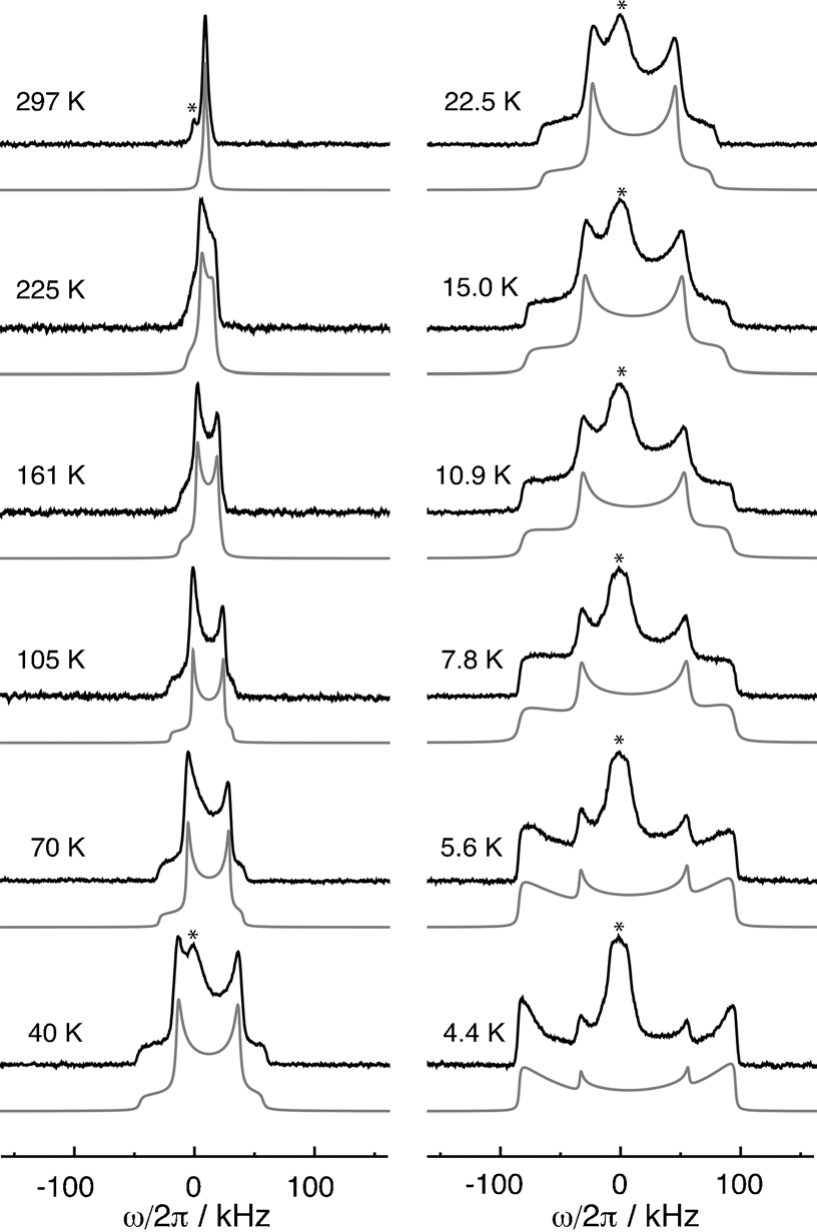
^1^H spectra of H_2_@C_70_ at 8.5 T. For each temperature the experimental spectrum is shown in black with the fitted line shape in gray. The asterisks denote signals from the protonated impurity. Each experimental spectrum is normalized to its own maximum intensity. All spectra are averages of 16 transients with an inter-scan delay between 10 and 20 times longer than the measured *T*_1_ for the endohedral hydrogen.

Figure 5 shows the ^1^H NMR spectra acquired at a range of temperatures, using the saturation-recovery solid-echo pulse sequence shown in Figure 3.

The 297 K spectrum (top left in Figure 5) is similar to the single-pulse spectrum in Figure 2, but with a greatly reduced impurity peak (indicated by the asterisk). This is because the 90°–90° echo sequence strongly suppresses the proton impurity signals, as discussed above.

The endohedral peak gets broader and starts showing a structure below 225 K. At 160 K the line shape develops into an asymmetric two-horn pattern with two shoulders. A symmetric two horn pattern is typical of powder spectra for randomly oriented isolated homonuclear spin pairs as originally observed by Pake.^28^ The spectral asymmetry is attributed to an axially symmetric chemical shift anisotropy interaction, collinear with the magnetic proton-proton dipolar interaction between the two protons. As discussed below, the spectra are consistent with a homonuclear dipole–dipole interaction that increases in magnitude with temperature, while the chemical shift anisotropy contribution is almost temperature-independent. As result the spectra become more symmetrical at low temperature when the dipole–dipole term dominates.

The impurity peak (indicated by an asterisk) decreases in amplitude (relative to the endohedral peak) over the temperature range between 230 and 40 K, only to reappear at lower temperatures. There are two reasons for this: 1) the relaxation time *T*_1_ of the endohedral protons is unusually short over this temperature range. This leads to an enhanced intensity of the endohedral proton peak relative to the impurity peak, since the proton magnetization of the impurity fails to recover fully after the saturation comb, while the rapidly relaxing endohedral proton magnetization recovers almost completely. 2) the endohedral proton peak becomes much broader at low temperatures, due to the strong intramolecular dipole–dipole coupling, while the width of the impurity peak does not have a strong temperature dependence.

The endohedral H_2_ lineshape at 15.0 K resembles closely the classical Pake form, with strong inner horns (due to dipolar interaction tensors with principal axes perpendicular to the magnetic field), and weaker shoulders (due to dipolar interaction tensors with principal axes parallel to the magnetic field). However, below 15.0 K, an unusual intensity perturbation is clearly seen. The “parallel” shoulders gain strongly in intensity as the temperature is decreased, at the expense of the “perpendicular” horns. At the lowest temperature of 4.4 K, the shoulders are so strongly enhanced that they form a pair of outer horns of greater amplitude than the inner horns of the normal Pake pattern. As discussed below, we attribute this effect to magnetic orientation of the C_70_ cages at low temperature.

### 2.3. Spin-Lattice Relaxation

The ^1^H nuclear spin-lattice relaxation was measured at each temperature by following the recovery of the NMR signal as a function of the delay *τ*_d_ using the sequence shown in Figure 3.

**Figure 6:**
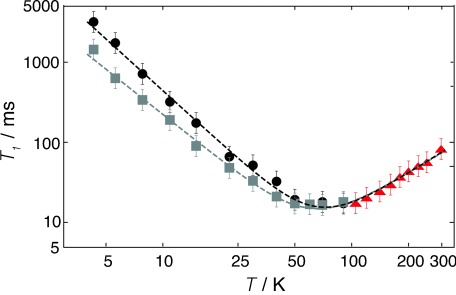
Temperature dependence of the ^1^*H* spin-lattice relaxation time *T*_1_ for H_2_@C_70_ at a magnetic field of 8.5 T, on a log–log scale. Triangles: relaxation time constant of the endohedral H_2_ (spectral integral) at temperatures *T*>100 K. Squares: relaxation time constant of the “perpendicular horns” at temperatures *T*<100 K. Squares: relaxation time constant of the “parallel shoulders” at temperatures *T*<100 K. Dashed lines: best fits of the relaxation time constants to the equation *T*_1_(*T*)=*AT*^*a*^+*BT*^*b*^. Grey dashed line: best fit to the perpendicular horn data, with *A*=16.1 s, *B*=0.02 ms, *a*=−1.9, *b*=1.4. Black dashed line: best fit to the parallel shoulder data, with *A*=61.0 s, *B*=0.02 ms, *a*=−2.1, *b*=1.5.

At temperatures below ∼100 K, the spin-lattice relaxation is clearly anisotropic. The magnetization recovery of H_2_ molecules encapsulated in C_70_ cages with long axes perpendicular to the field (the “horns”) is significantly faster than that of H_2_ molecules in C_70_ cages with long axes parallel to the field (the “shoulders”). In both cases there is a good fit to a single-exponential recovery curve. The *T*_1_ values for H_2_ encapsulated in cages with long axes parallel and perpendicular to the magnetic field are plotted separately against temperature in Figure 6. The perpendicular and parallel relaxation time constants converge at a temperature of ∼70 K.

At temperatures above ∼100 K, the spectral features are less distinct, and there is also overlap with the protonated impurity peak. The recovery of the complete spectral integral was analyzed in this regime, and was found to be biexponential. The fast-relaxing component was attributed to the endohedral hydrogen, and the slower component to the protonated impurity. The endohedral relaxation time constants are shown in Figure 6.

As shown in Figure 6, the *T*_1_ values for the endohedral H_2_ are roughly proportional to *T*^−2^ in the low-temperature regime, and proportional to *T*^+1.5^ in the high-temperature regime.

## 3. Discussion

### 3.1. Spatial Quantization

The endohedral H_2_ molecule is a quantum rotor confined to the interior of the C_70_ cage. The general features of the quantum dynamics of confined H_2_ in a symmetric potential have been discussed.^29^, ^30^ The position and orientation of the rotor is defined by the vector **R**, which describes the position of the H_2_ centre of mass relative to the centre of the cage, and the internuclear vector **r**. The Born–Oppenheimer quantum dynamics of the confined H_2_ molecule has six degrees of freedom: three to describe the location of the centre of mass, two for the orientation of the confined molecule, and one for the internuclear distance. The spatial quantum state of the confined *H*_2_ molecule may be described by a set of six quantum numbers. For example, an analysis of the infrared spectrum of H_2_@C_60_^12^ used the following quantum numbers: *v*, *N*, *L*, *J*, Λ, *M*_Λ_, where *v* is a vibrational quantum number, *N* is a translational quantum number, *L* is a quantum number for the quantized orbital motion of the molecule inside the cavity, *J* is a quantum number for the molecular rotation, and Λ, *M*_Λ_ are quantum numbers for the coupled rotational and orbital angular momentum. Since C_70_ has lower symmetry than C_60_, not all of these quantum numbers are good quantum numbers for the spatial eigenfunctions confined inside C_70_. Nevertheless, it remains true that the spatial Schrödinger equation for the confined molecule has six degrees of freedom. In the discussion below, a spatial stationary state is denoted Ψ_**n**_(**R**,**r**), where the spatial quantum numbers are denoted by the collective symbol **n**.

In addition, there are two quantum numbers *I* and *M_I_*∈{−*I*,−*I*+1…+*I*} for the nuclear spin angular momentum. Proton NMR spectra are generated by *ortho*-H_2_ molecules in the state *I*=1, which have odd values of the rotational angular momentum *J*.

**Figure 7:**
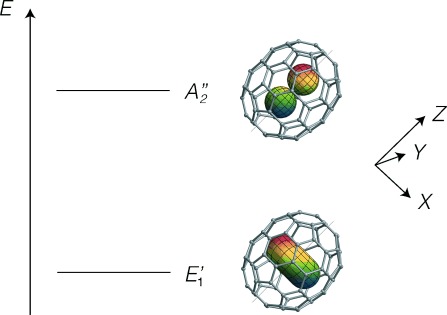
The two lowest energy levels of *ortho*-H_2_@C_70_ ordered after the experimental findings, see section 4.3.2. The labels *E*_1_′ and *A*_2_′′ indicate the irreducible representations of the group *D*_5*h*_ to which the levels belong. The wavefunctions of the confined hydrogen are represented by the torus-shaped and *p_z_*-shaped orbitals inside the fullerene, respectively. {*X*,*Y*,*Z*} denotes the cage axis system in which the *Z* axis is directed along the principal axis of C_70_.

The Schrödinger equation for H_2_ inside C_70_ has been solved numerically using a confining potential derived from a spectroscopically optimized carbon–hydrogen interaction.^19^ The symmetry and the degeneracy of the spatial energy levels conform to the irreducible representations of the symmetry point group *D*_5*h*_ of C_70_.^31^ At room temperature and below, only the *J*=1 states of *ortho*-H_2_ are significantly populated. The *ortho*-H_2_ *J*=1 ground state, which is triply degenerate in the case of H_2_@C_60_, is split in the case of H_2_@C_70_ into a non-degenerate *A*_2_′′ symmetric level, corresponding to a rotating hydrogen molecule longitudinally polarized with respect to the C_70_ long axis, and a doubly degenerate *E*_1_′ symmetric level, corresponding to transverse rotational polarization with respect to the C_70_ long axis. The spatial wavefunction of H_2_ in the *A*_2_′′ state has the form of a *p_z_* atomic orbital, oriented along the long axis of the cage (see Figure 7). The spatial wavefunctions of the degenerate *E*_1_′ states may be represented either as transverse *p_x_* and *p_y_* orbitals, or as complex superpositions of those orbitals, giving rise to torus-like complex wavefunctions (see Figure 7).

Numerical analysis of the spatial Schrödinger equation for H_2_@C_70_ predicts that the non-degenerate *A*_2_′′ level is ∼0.9 meV lower in energy than the doubly-degenerate *E*_1_′ level.^19^ However, as discussed below, the NMR results support the opposite ordering, with a doubly-degenerate ground level, and a non-degenerate upper level. The lowest energy levels of H_2_ in C_70_, and their associated wavefunctions, are shown with the experimentally supported energy ordering in Figure 7.

### 3.2. Spin Hamiltonian

The NMR spectrum is obtained from an effective spin Hamiltonian averaged over all the populated energy levels since lattice modes induce fast transitions between the molecular states with respect to the timescale of the spin interactions. The *D*_5*h*_ symmetry of the cage imposes uniaxial symmetry on the average chemical shift tensor and the dipole–dipole ^1^H–^1^H coupling tensor. The unique principal axes of both of these are tensors are parallel to the C_70_ long axis, which is assumed to subtend an angle *β* with respect to the applied magnetic field. In a powder sample, *β* is randomly distributed over the ensemble of C_70_ molecules.

The effective spin Hamiltonian for endohedral H_2_, in the high-field limit, is dependent on the orientational angle *β* of the C_70_ long axis with respect to the magnetic field, and on temperature. If the spin-rotation interaction is omitted (see discussion below), the spin Hamiltonian may be written as Equation (1):


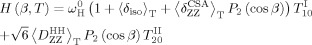
(1)

where the spherical tensor spin operators are given in terms of the nuclear spin angular momentum operators by Equations (2) and (3):


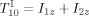
(2)


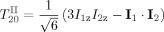
(3)

and *P*_2_(cos*β*)=1/2 (3 cos^2^*β*−1). The isotropic chemical shift of the endohedral protons is denoted by *δ*_iso_. The chemical shift anisotropy and proton–proton dipole–dipole coupling interactions are both described by traceless symmetric second-rank tensors. The *ZZ* components of these coupling tensors are denoted by 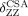 and 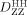, where the *Z*-axis denotes the long axis of the C_70_ cage. The angular bracket 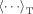 denotes the average over all thermally populated spatial quantum states, for example [Eq. (4)]:


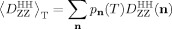
(4)

where *p*_**n**_(*T*) is the Boltzmann population of the spatial state with quantum numbers **n** at temperature *T*, and 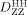
(**n**) is the dipole–dipole interaction tensor component for an individual spatial quantum state [Eq. (5)]:


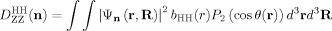
(5)

Here *θ*(**r**) is the angle between the internuclear vector **r** and the long axis of the fullerene cage, and *b*_HH_(*r*)=−(*μ*_0_/4 π)*γ*_H_^2^ħ*r*^−3^ is the proton–proton dipole–dipole coupling constant for a proton–proton distance *r*.

At high temperatures, a large number of spatial states are populated, while at sufficiently low temperatures, only the ground spatial state is populated. The low-temperature NMR spectrum is therefore a sensitive probe of the spatial ground state. The temperature-dependence of the NMR spectra may be used to test models of the excited spatial states, and their energies.

Equation (1) omits the spin–rotation interaction, which contributes strongly to the proton magnetic resonance spectra of H_2_ in molecular beams^32^, ^33^ and to proton relaxation in the gas and solution phases.^34^ The effects of spin–rotation interactions on the spectra of the confined H_2_ in the solid state have been discussed in ref. 35 where it is shown that the spin–rotation interaction would affect only the spectra in levels with *E* symmetry, resulting in lineshapes with a characteristic concavity at the centre of the spectra. The observation of spectral spin–rotation effects would require that transitions among the two degenerate states are slow on the spectral NMR timescale. No experimental NMR spectra have yet displayed unambiguous evidence of spin–rotation interactions at the time of writing. It must be assumed that rapid transitions between the spatial quantum states, induced by lattice fluctuations, suppress the spectral effects of spin–rotation interactions, even at cryogenic temperatures.

### 3.3. Lineshapes

The two single-quantum transition frequencies of *ortho*-H_2_ depend on the angle *β* between the C_70_ axis and the applied magnetic field **B**_0_ as in Equation (6):


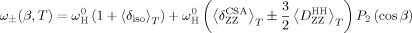
(6)

In the absence of line broadening, the NMR line shape in the frequency domain *s*(*ω*)=*s*_+_(*ω*)+*s*_−_(*ω*) is given by Equation (7)^36^:


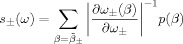
(7)

where 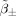 are the solutions of *ω*=*ω*_±_(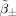,*T*). For isotropically distributed C_70_ cages, the probability density of *β* is given by Equation (8):


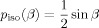
(8)

**Figure 8:**
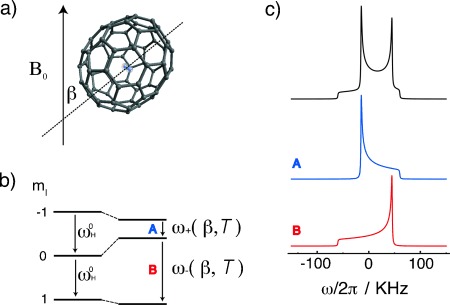
a) H_2_@C_70_ molecule with long axis at an angle *β* with the external magnetic field *B*_0_. b) ^1^H spin energy levels and the single-quantum transitions giving rise to the NMR spectrum. c) The powder NMR spectrum (asymmetric Pake doublet) and its two components, assuming uniaxial dipole–dipole coupling and chemical shift anisotropy tensors sharing the same principal axis system.

**Figure 9:**
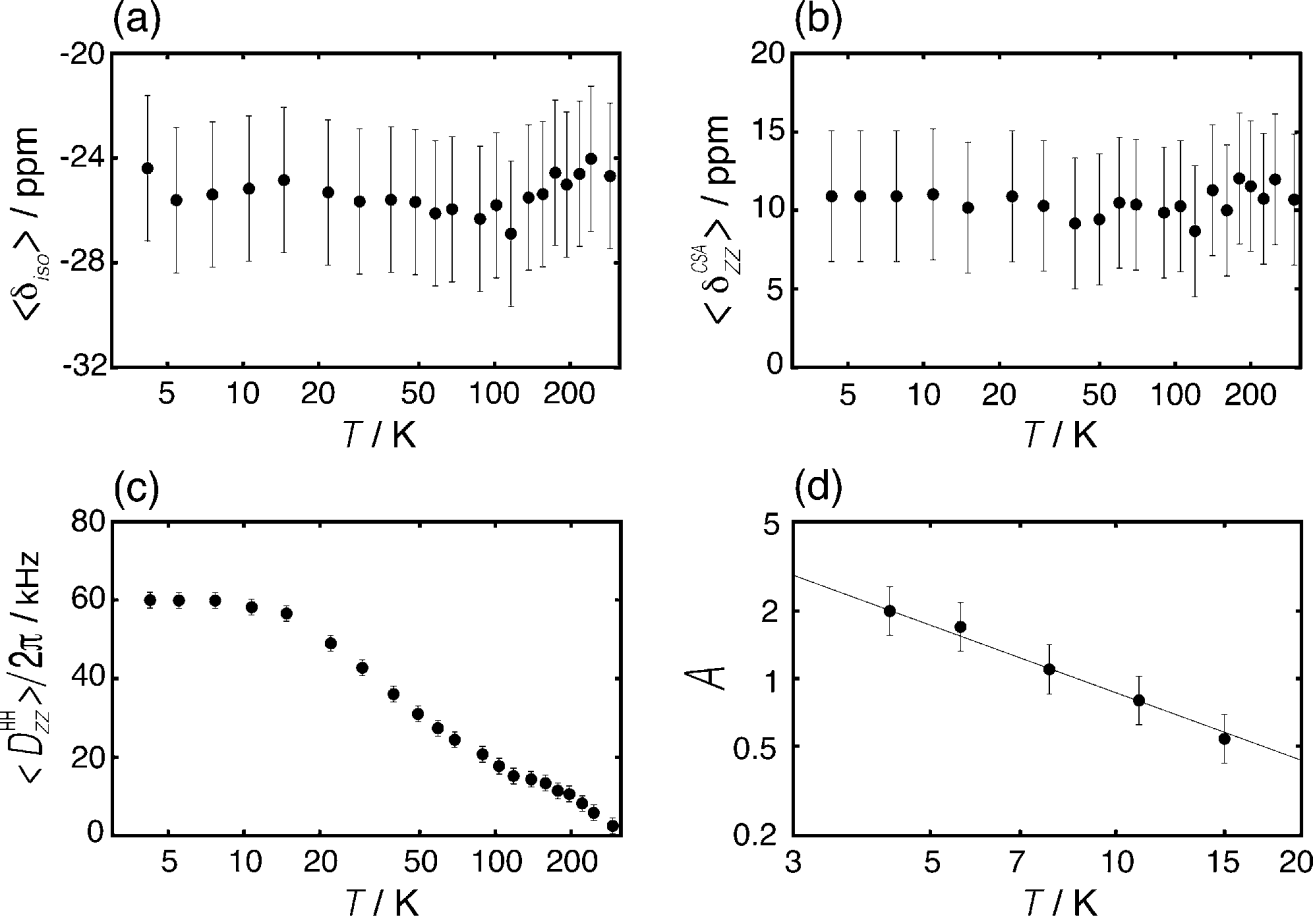
Temperature dependence of the spin interaction parameters a) 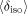, b) 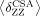, c) 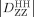 and d) the magnetic orientation parameter *A* from the fitting of experimental spectra in a field of 8.5 T. The gray line in (d) shows the function *A*(*T*)=8.7 K/*T*. All temperature axes are shown with a log scale. The vertical scale in (d) is also a log scale.

An isotropic orientational distribution is found to be sufficient to treat the proton spectra of H_2_@C_70_ at temperatures above ∼15 K. According to Equation (6), the chemical shift anisotropy is added to the dipolar constant for one single-quantum transition, and subtracted for the other. This gives rise to an asymmetric Pake-like doublet in powder samples (Figure 8). The horn–horn separation is 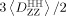, independent of the shift anisotropy 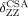. The frequency coordinate halfway between the two horns is 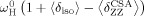. The fitted values of the spatial-average isotropic chemical shift 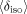, the spatial-average chemical shift anisotropy 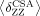, the spatial-average dipole–dipole coupling 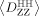, and their confidence limits, are plotted against temperature in Figure 9. The fitted lineshapes are shown below the experimental spectra in Figure 5.

The spatial-average isotropic shift 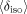 and chemical shift anisotropy 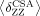 are both independent of temperature over the full range from 4 K to 297 K, within the confidence limits of the analysis. The spatial-average isotropic chemical shift and the shift anisotropy are given by 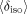
=−24.7±3 ppm and 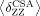
=10.1±4 ppm. The spatial-average dipole–dipole coupling 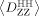, on the other hand, has a strong temperature-dependence above ∼15 K, falling steeply from ∼60 kHz at 15 K to <1 kHz at 297 K.

#### 3.3.1. Chemical Shifts

The values and the temperature independence of the parameters 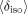 and 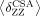 suggests that these chemical shift interactions are dominated by a source external to the H_2_ molecule. The experimental isotropic chemical shift of H_2_ in the gas phase is *δ*_iso_=7.40 ppm.^37^ For molecular hydrogen in the gas phase the chemical shift anisotropy can be estimated directly from the knowledge of the spin–rotation interaction coupling:^38^ 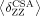
=1.6 ppm using the spin–rotation coupling from molecular beam studies.^39^ If the CSA were due to the H_2_ molecule itself, its magnitude would reduce in step with the dipolar coupling when the temperature is increased (Section 4.3.2).

We postulate, based on the measurements reported herein and previous literature studies of probes other than H_2_ in fullerene systems,^40^–^42^ that the electrons of the C_70_ cage generate the dominant contribution to both isotropic and anisotropic chemical shifts for the endohedral protons.

Figure 4 shows results of DFT chemical shielding tensor calculations within and just outside the C_70_ cage. Figures 4 a,b show the position dependence of the isotropic chemical shielding *σ*_iso_(**R**) while Figures 4 c,d show the relevant component of the chemical shift anisotropy tensor 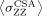
(**R**)=*σ_ZZ_*(**R**)−*σ*_iso_(**R**), where the *Z*-axis is parallel to the long axis of the cage. In order to compare with experiment, these shielding values were converted into chemical shift. After points outside the cage or within van der Waals contact of a carbon atom were excluded, the isotropic chemical shift (relative to the gas-phase molecular hydrogen) was found to be *δ*_iso_=−25.8 ppm, while the chemical shift anisotropy is 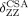
=8.7 ppm. These values represent the average of the chemical shielding over the van der Waals volume of the cage. The agreement of the calculated chemical shift anisotropy 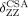
=8.7 ppm with the experimental value 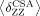
=10.1±4 ppm is gratifying.

The agreement of the calculated value of the isotropic chemical shift *δ*_iso_(**R**)=−25.8±0.5 ppm with the experimental value 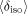
=−24.7±3 ppm is also encouraging. We also report excellent agreement with the result of −28.8 ppm reported for a helium-3 probe in ref. 40, but note that the agreement with our experimental value may be partly fortuitous, since the DFT calculation gives the additional chemical shift provided by the cage to whatever is inside, for an isolated C_70_ molecule in vacuum, while the experimental values are relative to tetramethylsilane in solution, and are measured in a bulk solid.

The most relevant aspect of the isotropic shift calculations is that the shielding inside the cage is predicted to be quite uniform, which agrees well with the observed temperature-independence of the isotropic chemical shift.

#### 3.3.2. Dipolar Couplings

The experimental values of the spatially-averaged dipole–dipole coupling 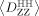 are strongly temperature-dependent, decreasing sharply at high temperature as more spatial quantum states are accessed. This corresponds to the quantum equivalent of motional narrowing. However, the dipole–dipole coupling parameter 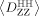 becomes temperature-independent below ∼10 K. This indicates that 1) the lowest energy level of *ortho*-H_2_ is separated from the next excited state by an energy corresponding to ∼10 K, and 2) the dipole–dipole coupling parameter in the spatial ground state is given by 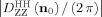
|=60±2 kHz, where **n**_0_ is the set of quantum numbers for the spatial ground state.

This value of the dipole–dipole coupling may be used to assign the spatial ground state. As shown by Tomaselli,^35^ the theoretical dipole–dipole couplings in the longitudinal *A*_2_′′ and transverse *E*_1_′ states are given by Equations (9) and (10):


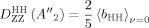
(9)


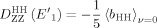
(10)

where 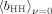 is the proton–proton dipolar coupling constant averaged over the ground-state vibrational wave function.^35^ If we assume that the ground state is *E*_1_′, then the experimental value of 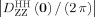
=60±2 kHz leads to the following estimate of the vibrationally averaged ^1^H–^1^H distance: 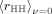
=73.8±0.8 pm. This agrees reasonably well with the ^1^H–^1^H distance of *r*_HH_=74.6 pm in free H_2_.^39^ The assignment of a *A*_2_′′ ground state would only agree with the experimental data if an unfeasibly long internuclear distance of 92.9±1.0 pm were assumed for the hydrogen molecule.

Our conclusion that the ground state of *ortho*-H_2_@C_70_ has symmetry *E*_1_′ is supported by recent infrared studies of the same system.^24^ However, numerical modelling of the five-dimensional quantum mechanics for endohedral hydrogen in C_70_, using an empirical Lennard-Jones potential, concluded that the ground state has *A*_2_′′ symmetry.^19^ A more refined description of the hydrogen–carbon interaction may be needed to match the experimental evidence.

#### 3.3.3. Magnetic Alignment of C_70_

The experimental results in Figure 5 indicate that the orientational distribution of the fullerene cages ceases to be isotropic at temperatures below ∼15 K. In order to interpret the low-temperature lineshapes, we postulate the following temperature-dependent probability distribution for the angle *β* between the long axes of the C_70_ cages and the applied magnetic field [Eq. (11)]:


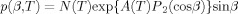
(11)

where *N*(*T*) is a normalisation constant. The sign of the parameter *A* determines the sense of the magnetic alignment: positive values favour orientation of the C_70_ long axes parallel to the field while negative values favour orientations perpendicular to the field. The isotropic distribution *p*(*β*)=*p*_iso_(*β*) is recovered in the limit of *A*=0. At this point, we allow an arbitrary temperature-dependence for the parameter *A*. Equation (11) is informed by the physical insight that each C_70_ molecule has a magnetic susceptibility tensor, with second-rank rotational properties.

We fitted the lineshapes below 15 K by varying the interaction parameters 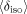, 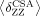, and 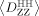 as well as the parameter *A*. The fitted lineshapes are shown below the experimental spectra in Figure 5. The fitted parameters, and their confidence limits, are shown in Figure 9. The magnetic orientation parameter *A* was found to have a linear dependence on inverse temperature, suggesting a thermal process associated with a magnetic energy Δ*E* [Eq. (12)]:


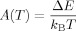
(12)

where *k*_B_ is the Boltzmann constant. The estimated value of the magnetic reorientation energy is Δ*E*/*k*_B_=8.7±0.3 K. Hence, at temperatures lower than ∼10 K, the long axes of the C_70_ cages align significantly along the magnetic field, causing a strong enhancement of the Pake pattern “shoulders”, at the expense of the inner “horns”.

The alignment of the C_70_ cages with the magnetic field could involve the anisotropy Δ*χ* of the magnetic susceptibility tensor. For isolated single molecules this would lead to a magnetic interaction energy given by Δ*E*=Δ*χB*_0_^2^/2 *μ*_0_ where Δ*χ* is the susceptibility anisotropy.^43^ However, the computed value of Δ*χ*=6.3×10^−33^ m^−3^ corresponds to an orientational energy of Δ*E*/*k*_B_=0.01 K in a magnetic field of 8.5 T. We conclude that the magnetic anisotropy of individual C_70_ molecules is about two orders of magnitude too small to explain the observed effect. The observed magnetic orientation must therefore be a cooperative effect involving many neighbouring C_70_ molecules. This could involve the magnetic reorientation of microscopic C_70_ domains, or possibly the reorientation of entire crystallites.

Alignment of molecules with respect to an applied magnetic field is well-known in the solution NMR of biomolecules, where it is used to assist molecular structure determination.^44^ Cooperative molecular alignment in a magnetic field is also well-known for liquid crystals.^45^ The phenomenon observed here seems to be of the same kind, but occurs at an extraordinarily low temperature. We are not aware of an analogous physical phenomenon in this temperature regime.

We have also performed preliminary experiments at a higher magnetic field of 14.1 T. Contrary to expectations, the magnetic ordering effects were found to be weaker than those shown in Figure 5. This requires further investigation, but it is possible that the degree of magnetic alignment in H_2_@C_70_ depends on the sample preparation (type and amount of occluded solvents, homogeneity, crystallinity) and possibly on the thermal history as well. More investigations are in progress.

### 3.4. Spin–Lattice Relaxation

Nuclear spin relaxation in molecular hydrogen is determined by the modulation of the spin–rotation and of the dipolar Hamiltonians induced by the interaction of the molecular angular momentum **J** with the lattice.^34^, ^46^ Spin relaxation is effective when the temperature-dependent correlation time *τ*_c_ for the fluctuating interactions matches the Larmor frequency of the spins 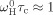.^47^ *T*_1_ is long at low temperatures when 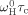
≪1 or at high temperature when 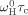
≫1. The experimental observation of a single minimum 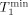
=14±3 ms at *T*=70 K (see Figure 6) supports a model with a single mechanism over the full temperature range, and with a monotonic dependence of the correlation time *τ*_c_ on temperature.

Fedders derived explicit formulae for the theoretical ^1^H spin relaxation rates for *ortho*-H_2_ trapped into a solid at sites with cubic, axial and low symmetry.^46^ The derived expressions for 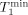 do not depend on the correlation time *τ*_c_ and are independent of the model used to describe the interaction with the lattice. For the magnetic field *B*_0_=8.5 T used in our experiments, the Fedders theory predicts 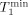
=5 ms in the case of a uniaxial H_2_ environment.^46^ This is almost three times smaller than the observed value of 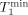. This observation is in contrast to the study of *ortho*-H_2_ trapped in rare gas and *para*-H_2_ matrices, where a good match with the Fedders theory was observed.^48^

We do not understand the reasons for the observed deviations from the Fedders theory at the present time. The strongly anisotropic environment of H_2_ in C_70_, which gives rise to resolved spectral features, could play a role. It is also possible that cross-relaxation with the slowly-relaxing impurity protons artificially lengthens the H_2_ relaxation time. A phenomenon of this type has been observed in studies of relaxation for H_2_ encapsulated in an open-cage fullerene containing protonated exohedral groups.^8^ High purity powders are in preparation in our laboratory in order to address these issues.

## 4. Conclusions

**Figure 10:**
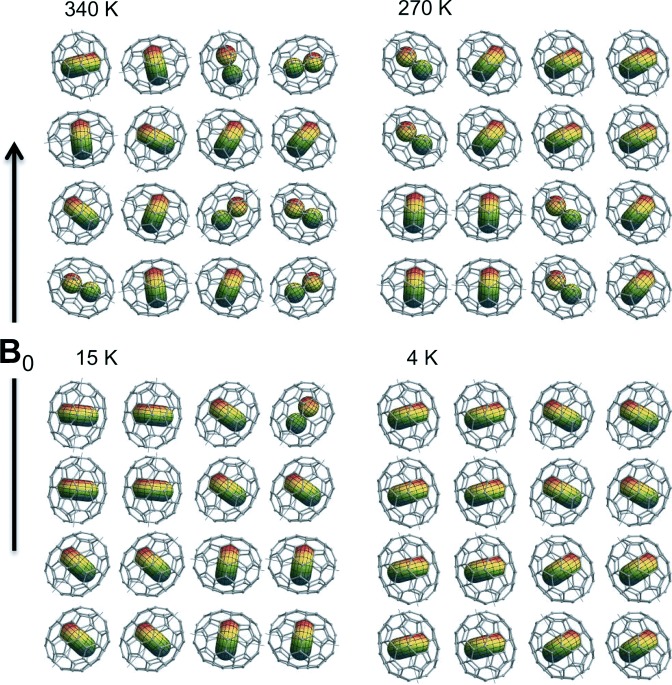
Pictorial representation of H_2_@C_70_ in the solid phase, according to the proton NMR data. Two spatial quantum states for the endohedral H_2_ molecules are shown: *A*′′_2_, which is shown as a *p_z_*-orbital shape, and the doubly degenerate *E*′_1_ state, which is shown as a torus. At 340 K the C_70_ molecules are free to orient and the instantaneous orientational distribution is isotropic. Below the orientational ordering phase transition at 270 K the cages lose their rotational freedom and domains of coaxial molecules are formed. The correlated domains are represented in the Figure by 2×2 blocks. At 15 K the H_2_ molecules are all found in the *E*′_1_ ground state, and at 4 K the C_70_ domains orient along the magnetic field.

The physical picture of H_2_@C_70_ that emerges from these investigations is summarised, in a highly simplified form, in Figure 10. At high temperature *T*>340 K (top left), the C_70_ cages rotate isotropically and the solid phase has cubic symmetry.^1^, ^49^, ^50^ In addition, the endohedral H_2_ molecules explore a wide range of accessible quantum states (Figure 10 shows only the *A*′′_2_ and *E*′_1_ states, populated in the degeneracy ratio of 1:2, for simplicity). This gives rise to a greatly reduced dipole–dipole interaction, corresponding to classical motional averaging of the dipole–dipole coupling through isotropic molecular tumbling.

On lowering the temperature, isotropic reorientation is replaced by a fast tumbling or a precessional motion of the cages along a preferred crystal axis.^50^ Other studies suggest that the plastic cubic phase with almost isotropic rotation of the cages axis persists even below room temperature.^51^–^53^ In any case it has been recognized that below 270 K (top right), correlations develop between the orientations of neighbouring C_70_ cages. The Figure shows the C_70_ orientations organised in four 2×2 blocks.

At a temperature of 15 K (lower left), all H_2_ molecules are in the *E*′_1_ ground state. This generates a Pake pattern with a dipole–dipole coupling constant of ∼60 kHz. Although neighbouring C_70_ molecules have correlated orientations, there is no net orientation with respect to the magnetic field.

At a temperature of 4 K, the C_70_ molecules partially align so that their long axes are parallel to the magnetic field.

Figure 10 should not be taken too literally. For example it is likely that the C_70_ cages align cooperatively in small domains, or perhaps that entire crystallites align with the magnetic field. Furthermore, it is yet not known whether the magnetic alignment behaviour is also exhibited by empty C_70_ cages, or whether the endohedral H_2_ molecules somehow influence the behaviour of the cages and their mutual interaction. Although there is a possibility that a phase transition occur at ∼15 K, the observation of alignment effects over a considerable temperature range suggests that this is not the case. In addition, even if there were a phase transition, there would still need to be a mechanism leading to macroscopic alignment of the cages, in which the magnetic susceptibility anisotropy is still implicated.

The low-temperature magnetic alignment of C_70_ cages requires further investigation, in order to elucidate whether the thermal and physical history of the sample, or the presence of impurities, play a role, or whether hysteresis is observed with respect to temperature. Higher-purity samples are currently in preparation in our laboratory for the purpose of such studies.

The low-temperature NMR data show that endohedral H_2_ molecules may act as “spies”, allowing the proton NMR spectra to report on the low-temperature behaviour of the fullerene cages—in a similar way to muon spectroscopy.^50^–^53^ This might be of use in other contexts as well, such as the study of fulleride superconductivity.^54^, ^55^
